# Social Networks, Health Information Sharing, and Pandemic Perceptions among Young Adults in Hawai’i during the COVID-19 Pandemic

**DOI:** 10.3390/ijerph192416833

**Published:** 2022-12-15

**Authors:** Michael M. Phillips, Rosana Hernandez Weldon, Anam Maniar, Uday Patil, Uliana Kostareva, Joy Agner, Julia Finn, Tetine Sentell

**Affiliations:** 1 Office of Public Health Studies, Thompson School of Social Work & Public Health, University of Hawai’i at Mānoa, Honolulu, HI 96822, USA; 2 Nancy Atmospera-Walch School of Nursing, University of Hawai’i at Mānoa, Honolulu, HI 96822, USA; 3 Chan Division of Occupational Science and Occupational Therapy, University of Southern California, Los Angeles, CA 90089, USA

**Keywords:** social networks, distributed health literacy, health literacy, COVID-19, native Hawaiian, Pacific islander, Filipino

## Abstract

Limited information exists about social network variation and health information sharing during COVID-19, especially for Native Hawaiians (NH), Other Pacific Islanders (OPI), and Filipinos, who experienced COVID-19 inequities. Hawai’i residents aged 18–35 completed an online survey regarding social media sources of COVID-19 information and social network health information measured by how many people participants: (1) talked to and (2) listened to about health. Regression models were fit with age, gender, race/ethnicity, chronic disease status, pandemic perceptions, and health literacy as predictors of information sources (logistic) and social network size (Poisson). Respondents were 68% female; 41% NH, OPI, or Filipino; and 73% conducted a recent COVID-19 digital search for themselves or others. Respondents listened to others or discussed their own health with ~2–3 people. Respondents who talked with more people about their health were more likely to have larger networks for listening to others. In regression models, those who perceived greater risk of acquiring COVID-19 discussed their health with more people; in discussing others’ health, women and those with chronic diseases listened to a greater number. Understanding young adults’ social networks and information sources is important for health literacy and designing effective health communications, especially to reach populations experiencing health inequities.

## 1. Introduction

As social beings, our health is interwoven within the networks of those with whom we associate and the individuals with whom they associate [1]. These social networks can directly and indirectly impact health outcomes through a variety of pathways, including interpersonal engagement, social norming, and practical support, as well as access to resources and information [1,2].

The coronavirus pandemic (COVID-19) has been accompanied by an “infodemic,” defined by the World Health Organization as “too much information including false or misleading information in digital and physical environments during a disease outbreak” [3]. An infodemic can cause not only uncertainty about health risks but mistrust in public health authorities as well [4,5]. Social networks have been influential sources of health information throughout the COVID-19 pandemic, a time of considerable uncertainty, conflicting guidance, changing policy, and dynamic scientific discovery [6]. 

Young adults have distinct social networks and information pathways. The digital information ecosystem, especially including social media, is of relevance for this population [7]. Young adults had high social media use before COVID-19, which only increased during the pandemic [8]. Despite widespread recognition of increased use of social media, limited information exists about detailed variation in social networks and health information sharing among young adults during COVID-19. This is especially true for Native Hawaiian (NH), Other Pacific Islander (OPI), and Filipino populations who have experienced deep COVID-19 inequalities [9,10,11]. 

NH, OPI, and Filipino populations have been considered “priority populations” in public health planning and action in the state of Hawai’i, USA [12,13]. This designation underscores the importance of ensuring that public health policy and practices are implemented with specific consideration of how to address the health inequities experienced by NH, OPI, and Filipino communities, ideally by engaging with community and building on community strengths [9]. A disproportionate number of NH, OPI, and Filipinos are affected by chronic diseases, e.g., asthma, diabetes, hypertension, and heart failure [14,15,16]. Even prior to the COVID-19 pandemic, higher rates of acute care, preventable hospitalizations and chronic conditions were found for NH, OPI and Filipino populations in Hawai’i [17,18]. Furthermore, young adults within these populations have been found to have high rates of chronic disease [18], and those who have chronic conditions are particularly vulnerable to complications due to COVID-19 [19]. Understanding health communication pathways is important for building trust and engagement in public health messaging, which is profoundly understudied at all ages in NH, OPI, and Filipino populations, including in young adults.

Building public health and health care programming and policy from community strengths and knowledge is critical to achieve goals of health equity [9]. NH, OPI, and Filipino communities have distinct and diverse histories, languages, and cultures, but also share some characteristics relevant to public health planning. For instance, communal perspectives of health and well-being are strong cultural values [20,21,22]. NH, OPI, and Filipino populations are also more likely to live in intergenerational homes than many other groups in Hawai’i, which is both a risk factor for COVID-19 exposure to be considered in public health planning [23,24,25], and also a potential resource for public health messaging reach across age groups [26]. Understanding health information sharing behaviors in social networks, an important factor in health communication, is paramount to understanding health literacy (HL) and to ensure health equity.

This study was grounded by the social-ecological model (SEM), with a focus on the individual and interpersonal aspects to inform policy decisions and help to set priorities in post-pandemic planning and public health practice [27]. Particularly, we performed a descriptive analysis of the intersections between these two levels of the model, especially examining interpersonal factors like social support and social ties, which have been linked to health outcomes and may vary by individual factors (e.g., gender, race/ethnicity, type of work, educational attainment) [28]. 

Overall, our objective was to describe and better understand the health information pathways of young adults in the state of Hawai’i, considering priority populations for achieving health equity. Additionally, we sought to understand the role of other potential factors that might play a role in health-related outcomes. For example, women tend to be family health caregivers for both aging parents and children within the household and thus might be expected to provide advice or listen to others’ health concerns [29]. Gender differences, as well as other socio-demographic characteristics, are important to consider in the social networks and health information ecosystems of young adults across distinct communities. 

Our specific research questions were: Are socio-demographic factors and health literacy associated with different facets of health information networks? Do individuals discuss health concerns with others or listen to others? If so, who tends to share or listen, and how big are their networks for sharing? How are these factors associated with COVID-19 perceptions of risk? Do they search for health-related information linked to COVID-19? If so, for whom? What online digital sources are they using for health-related information regarding COVID-19? Answering these questions can build a knowledge base for public health programming to support health equity and effective health information dissemination. 

## 2. Materials and Methods

### 2.1. Recruitment and Procedures

Participants were recruited to complete an online survey in Spring 2021 through a Qualtrics-managed online research panel. Survey respondents were pre-screened based on eligibility, which included being: (a) able to read and understand English, (b) a resident of Hawai’i or U.S. affiliated Pacific (Guam, American Samoa, Northern Mariana Islands), and (c) between the ages of 18 and 35 years old. Participation in this study was anonymous. Respondents were not asked for name or contact information at any point. On average, the survey took about 5–10 min to complete. Participants received small incentives of <$15 value through the Qualtrics research panel. 

### 2.2. Sample

For this study, we focused on participants in the state of Hawai’i because the response for U.S. affiliated Pacific islands was small (*n* = 7). Thus, the study sample size included 324 respondents, all Hawai’i residents. 

### 2.3. Overall Questionnaire

The battery of survey items was presented online through Qualtrics and targeted multiple topics. All items were self-reported, and some items were only completed based on prior responses utilizing skip-logic. COVID-19 questions were based on the University Students’ COVID-19 Health Literacy Survey [30] developed as part of the interdisciplinary COVID-HL network launched in February 2020 as an open science and research community. Other items were drawn from other established measures, including the eHEALS [31], social network questions [32], and self-reported health literacy measures [33]. The online battery of survey items was tested by Qualtrics for quality assurance and, during administration, had active checks for attention or understanding. All responses had to meet quality standards to be considered complete.

#### 2.3.1. Demographics 

Demographic items were collected, including: (1) age; (2) gender identity: Male, Female, Other (Non-binary/Third gender, Transgender male, Transgender female, Gender variant or Non-conforming); (3) race and ethnicity: Filipino, Native Hawaiian, Other Pacific Islander (Samoan, Tongan, Chuukese, Marshallese, Palauan, Chamorro/Guamanian, Fijian, Tahitian), Japanese, Chinese, Other Asian (Korean, Vietnamese, Asian Indian, Other Asian), Latino/a (Latinx, Mexican, Hispanic, Other Hispanic, Portuguese), Caucasian, Black/African American, Native American, Other, and Refused/Don’t know/Not sure; (4) if respondents marked more than one race and/or ethnicity for the previous question, they were asked which best represents their identity (based on same categories above for the race and ethnicity question, and this answer was used as for the race/ethnicity categorization; (5) disability status: Yes, No; (6) chronic condition lasting or expected to last at least 6 months: Yes, No; (7) highest level of educational attainment: Less than high school (Never attended school/Only kindergarten, Grades 1–8/Elementary, Grades 9–11/Some high school), Grade 12 or GED/High school grad, Some College (College 1–3 years/Some college/Tech), Bachelor’s degree/BA or BS, Graduate degree (MA, MS, PhD), and Don’t know, Refuse to answer. Given an expectation of variation across populations, we also asked household size and how many members of the household were under 18 and over 65 years of age.

#### 2.3.2. Social Network for Health

Two items were included to address size and function of respondents’ social networks for sharing important health-related matters. The questions asked individuals to report how many people in the last month with whom they discussed their own health and how many people they listened to about important health matters. 

#### 2.3.3. Online Digital Activity

To capture different types of online digital activity (ODA), respondents were asked about their past month’s searches and what sources they have been using recently for COVID-19 information. Two questions were utilized to frame respondents’ online digital activity when exploring sources of information for COVID-19 and related topics. The first question asked whether respondents purposefully searched the internet for COVID-19 information or related topics, and if so, for whom? If they searched for others, a follow-up question was asked to identify the “for whom” portion (*Parent*, *Grandparent*, *Child*, and/or *Other*). A second ODA question asked respondents what digital media outlets they utilized for locating COVID-19 and related information, with an opportunity to select all outlets that applied (*Facebook*, *Instagram*, *LinkedIn*, *Medium*, *Quora*, *Reddit*, *Snapchat*, *Stack Exchange*, *TikTok*, *Tumblr*, *Twitter*, *YouTube*, *4chan*, and/or *Other*). For the *Other* option, they were asked to complete a fill-in response. 

#### 2.3.4. Health Literacy

A modified version of the Single Item Literacy Screener [34] was used to measure HL. Participants self-reported on a 5-point rating scale (*Never*, *Rarely*, *Sometimes*, *Often*, *Always*) to the question, “How often do you need help to read instructions, pamphlets, or other written material from your doctor or pharmacy?” The responses were further dichotomized based on prior conceptualization [35] of high HL (*Never*, *Rarely*) and low HL (*Sometimes*, *Often*, *Always*).

#### 2.3.5. Perceptions of COVID-19

Two questions assessed respondents’ perceptions of the pandemic with a focus on their likelihood of catching COVID-19 and the impact on their lives if they did catch it. The first question regarding perceived susceptibility asked, “What are the chances of getting COVID-19?”, rated on a 4-point scale (*High*, *Medium*, *Low*, *No chance*), which was conceptualized for analyses as unlikely (*No chance* or *Low*) or likely (*Medium* or *High*). Next, respondents were asked, “How would getting COVID-19 affect your life?” to assess perceived impact, with the following rating scale (*This would make me: Very sick*, *A little sick*, and *This is not a big deal to me*), which were conceptualized as major impact (*Very sick*) or minor impact (*A little sick* or *This is not a big deal to me*) for analyses. 

### 2.4. Analysis Plan

Quality checks were completed on the final dataset, which included survey completion time, free-response items, and compliance to a quality-check question midway through the survey. Outliers were evaluated based on boxplots and using 1.5 + Inter-quartile Range (IQR) at the 75th percentile; they were explored for two outcome variables related to the number of individuals in participants’ social support network with whom they discussed health-related topics (i.e., their own health and the health of others). Two outliers were removed for the variable *discussion of own health* and four were removed regarding the variable *discussion of others’ health*.

#### 2.4.1. Descriptive Statistics

First, the sample was descriptively evaluated along with a sample breakdown by chronic disease status. Next, we created a cross tabulation to descriptively examine the question: How many searched for health information online and for whom? We looked by gender (*male*, *female*), race/ethnicity, educational attainment level (*up to a HS degree/GED*, *Some college*, and *BS/BA or graduate degree*), chronic disease status, disability status, and HL. We also considered social network size for discussion of health (*discussion of own health*, *discussion of others’ health*) and household factors (e.g., size, number under 18, and number over 65) by gender, race/ethnicity, HL, chronic disease, and disability status. 

#### 2.4.2. Inferential Statistics

Two Poisson regression models were fit for each of the social support network variables (*discussion of own health, discussion of others’ health*) based on the count nature of these outcomes utilizing an entry method based on the exploratory nature of the study. When evaluating the sample size of subsample groups, we eliminated a few subsamples for purposes of the analyses. For example, for gender there were seven cases across the categories other than female or male. Also, for race/ethnicity, the groups with a large enough sample size for inclusion in the regression models were NH, Filipino, Japanese, Chinese, and White. Both regression models included predictors for age, gender (*male*, *female*), race/ethnicity, chronic disease, pandemic perceptions (whether it would affect their life and chances of catching it), and health literacy. Utilizing the same predictors, a multivariable logistic regression was fit to explore those that used Facebook/Instagram as a source of information for COVID-19 or health-related information. 

## 3. Results

### 3.1. Demographics

Descriptive statistics are reported in Table 1. The final sample included 324 individuals between ages 18–35 years with a mean age of 26.4 (SD = 5.48, Mode = 32). There were more than twice as many female (*n* = 219; 67.6%) compared to males (*n* = 98; 30.2%), and seven individuals identified as one of the other options for gender (2.2%). 

When considering race and ethnicity, a total of 32% of the sample selected more than one race or ethnicity and was categorized based on the follow-up identity question. Following this categorization, close to one-fifth of the sample identified as NH (19.4%), 18.2% as Filipino, 22.8% as White, 5.6% as Latinx/Hispanic and 3.1% as OPI. One-quarter of the sample identified as either Japanese (14.8%), Chinese (6.2%), or other Asian (4.6%). In educational attainment, 66.6% of the sample had some college or above (with 37.6% attaining a college or graduate degree).

### 3.2. Social Network for Health Information 

On average, the young adults surveyed were discussing their own health with 2–3 people (overall M = 2.18, SD = 2.95) and listening to roughly the same number of people talk about health issues (overall M = 2.49, SD = 3.88). However, there was greater variation in the number of people respondents listened to. In addition, the mode for the number of people with whom respondents shared their own health concerns was 0, and the mode for the number of people respondents listened to was 2. We found that respondents who talked with a greater number of individuals about their own health were also more likely to have larger networks for listening to others, with a significant positive correlation between the two (r (317) = 0.614, *p* < 0.001). Notably, over 28% of those shared their health concerns with no one, and 20% had no one share health concerns with them. See Table 2 for a descriptive breakdown by socio-demographic, social context within the household, and related factors. 

#### 3.2.1. Predictors Related to Size of Network to Discuss Own Health

A Poisson regression model was fit (Table 3) with age, gender, race/ethnicity (NH, Japanese, Chinese, Filipino, and White), chronic disease, COVID-19 perceptions (whether it would affect their life and chances of catching it), and health literacy as predictors of the number of people with whom respondents discussed their own health (*discussion of own health*). A significant association was found for the pandemic perception related to their perceived chances of catching COVID-19 (*p* < 0.001). Specifically, individuals perceiving a greater chance of catching COVID-19 was associated with discussing their own health with a greater number of individuals (IRR = 1.447; 95% CI: 1.212, 1.728).

#### 3.2.2. Predictors Related to Size of Network to Discuss Others’ Health 

A Poisson regression model was fit (Table 4) with age, gender, race/ethnicity (NH, Japanese, Chinese, Filipino, and White), chronic disease, COVID-19 perceptions (whether it would affect their life and chances of catching it), and health literacy as predictors of the number of people respondents listened to regarding health topics (*discussion of others’ health*). A significant association was found for gender and chronic disease status (Table 4). The rate for females (IRR = 1.249; 95% CI: 1.029, 1.525) and those with a chronic disease (IRR = 1.510; 95% CI: 1.203, 1.880) tended to be greater for the number individuals they listened to regarding health topics. However, it is important to note that a small proportion of the population self-reported a chronic disease (*n* = 41, 12.7%). No significant differences were found for race/ethnicity (Japanese referent group).

### 3.3. How Many Are Searching for Health Information Online, and for Whom?

As can be seen in Table 5, in relation to online searches regarding COVID-19 information in the past month, slightly more than a quarter of individuals stated that they had not conducted any searches (27.5%), and 27.8% only searched for themselves. Another 10% searched solely for information for others, and the remaining 35% searched for information for both themselves and others. Of the 45% that searched for information only for others or for themselves and others, a follow-up question was asked about whom they were searching COVID-19 information for in regard to the others. The categories were not mutually exclusive, thus 12% marked some combination of the 4 choices (*Child*, *Parent*, *Grandparent*, *Others*). *Parents* (*n* = 66) or *Others* (*n* = 64) were the top two choices selected by the majority; *Grandparents* (*n* = 25) and *Children* (*n* = 17) were not as frequently the focus of their COVID-19 searches. 

### 3.4. Digital Media Outlets

The majority (99.7%) of the sample utilized some form of digital or social media to find information about COVID-19 or related topics. The two main digital media sources of COVID-19 information for this group of young adults were Facebook (63%) and Instagram (58%), both owned by Meta. Twitter (29.6%) and TikTok (30.6%) were each used by almost one-third, and Reddit (20.5%) and Snapchat (22.5%) were slightly more than one-fifth. Another popular source of digital information was YouTube (54.3%). Lastly, respondents were allowed to select and fill in for “other” sources (8.6%), with several of the open-ended responses listed as local and national news outlets. 

With the majority (81.2%) using Facebook, Instagram, or both, these two categories were combined into one outcome variable. A multivariable logistic model was conducted to assess predictors of Facebook/Instagram use (*yes*, *no*) as a source of COVID-19 and health-related information. There was no difference for age, gender, chronic disease status, pandemic perceptions, or HL. However, there were differences by race/ethnicity. With Japanese as the race/ethnicity referent group, use of Facebook/Instagram by NH (OR = 2.86; 95% CI: 1.05, 8.37) and Filipinos (OR = 2.96; 95% CI: 1.03, 9.31) was significantly higher, but was not significantly different between Chinese or White respondents. Holding other factors constant, NH and Filipino respondents were significantly more likely, close to triple the odds, to use Facebook/Instagram for COVID-19 and health-related information compared to Japanese respondents. 

## 4. Discussion

Almost all the young adults in this study used digital media and social media to find health-related information, particularly to find COVID-19 information during the pandemic. These young people were looking for information not only for themselves but also for others. Digital interactions, through the advent of web 2.0, have allowed individuals to engage with friends, family, peers, acquaintances, and strangers in ways never imagined and almost constantly. Even as access to technology has become more widespread [36,37], the digital divide between those with and without access to the internet has continued to exist. However, there are also pattern differences for digital information retrieval and platform use [38] when searching for health-related information. Facebook and Instagram tend to be heavily utilized by young adults as sources of information, with high use noted among NH and Filipino respondents (comparatively to Japanese). This information can be leveraged in public health interventions targeting young adults and when considering the potential impact of the infodemic, including ensuring that trusted, culturally-relevant health information for NH and Filipino youth is found on Facebook and Instagram.

This descriptive study found that the majority of young adults living in Hawai’i were engaging with their social networks to have conversations about COVID-19 and important health information. Respondents who talked with more individuals about their own health were also listening to a greater number of people, yet women were more likely to be listening to others’ health concerns compared to men. Others have also found a greater number of women providing health advice or listening to others’ health concerns within a family unit as the primary family health caregiver [29], and this aligned with our respondents’ listening patterns.

Although this engagement of sharing health information likely includes listening to others’ personal stories, and potentially sharing personal stories or inquiries, respondents only reported discussing their own health with an average of 2–3 people and listening to roughly the same. Additionally, a fairly high proportion of respondents stated that they did not discuss their health with anyone. This suggests that young adults may conceptualize listening and sharing health information differently in different contexts and via different platforms. This could be the case due to high social media use for finding health information that must not have been perceived as a way of discussing or listening to others’ health information. Future qualitative research could be used to probe these differing facets of young adults’ social networks and to reflect upon their experiences during the pandemic in greater depth.

The nexus of gender, ethnicity, and culture is important to consider in this work and will provide fruitful areas of future study [39]. These are critical issues in a multicultural state like Hawai’i [40]. There is a need to continue to understand how individuals navigate digital health spaces and the social context in which they spend their time based on the intersectionality of one’s identity. Social networks can play a crucial role in individuals’ health decisions and care management generally and specifically in NH, OPI, and Filipino populations [32,41]. Research has found that Filipino immigrants in Hawai’i have strong health care access due to social networks [42], while other studies in Hawai’i have found that men had fewer health discussion partners in their networks [41] and that wives and daughters play critical roles in social networks for health care among those with a chronic disease [32]. Men are also less likely to go to the doctor, especially when they are working age [43]. Additionally, social networks of older adults may be more homogenous than younger people [39]. 

Interpersonal relationships can protect individuals from negative influences on their health [44]; however, not all social relationships have a positive influence on health information or health behaviors, and social networks can also increase health risks. Tight networks can be supportive but might also have a social cost if myths or misinformation are shared within them. Indirect impact or spillover effect [45] within social networks can affect the implementation of protective health behaviors or the likelihood of engaging in behaviors that put individuals’ health at risk. For instance, infectious diseases and vaccine uptake do not spread through societies evenly but are more concentrated in some communities than others; substance use tends to be higher amongst those with individuals in their network that also use; and political messages have been shown to influence information-seeking and real-world behaviors (e.g., voting behavior) within social networks [46], particularly in newer digital spaces. With the polarization of views during COVID-19, there is the potential for siloed clusters within an individual’s network that reinforce a shared, potentially misinformed, narrative. These “echo chambers” are prevalent in social media spaces [47] and may influence individuals’ perceptions of the pandemic and lead them to engage in health behaviors that may or may not put them or others in their network at greater risk. 

For individuals to make informed decisions about their health and the health of others, digital HL is critical to ensure understanding of the value and veracity of materials posted in digital spaces. There is a need for greater digital HL education to help individuals critically evaluate the trustworthiness of health information they receive and share on the internet and within their social networks and for policy to regulate the trustworthiness and safety of information shared on the internet. Strength-based social media efforts by and for youth populations across cultures and languages are particularly important [26]. 

### Limitations

The means of data collection, i.e., through an online survey in English, could have impacted the types of respondents recruited to complete the survey, their response to the Single Item Literacy Screener as a proxy for health literacy, and their propensity to use digital sources for health information seeking. The sample might have included individuals with higher digital literacy and greater access to digital spaces. Additionally, there was limited variability in our sample when utilizing a one item proxy for health literacy that focuses on identifying individuals with limited reading ability who need assistance with health-related materials. One item as a proxy for all facets of a complex construct like health literacy is limiting and was based solely on a personal health literacy perspective. We also assessed individuals’ propensity to use digital sources for health information but did not assess their ability to make informed decisions based on these sources.

We did not consider all aspects of risk. An early global assessment regarding the impact of COVID-19 on 30-day mortality found greater levels of income inequality to be a consistent factor [48]. This influenced the poorer health outcomes that have been consistently related to health disparities across varying groups within society. Future research should examine the intersectionality of these vulnerabilities with information-seeking and sharing behaviors to tailor health communication interventions to highly vulnerable groups.

Additionally, several components of health-sharing behaviors and social networks were not included in this sample. The perspectives of health discussion partners on COVID-19 may have impacted the perspectives of the young adults in our sample. Future research should gain further details about these networks, including the relationship and perceived perspectives of the network members. Furthermore, since social media has risen as such a common source of health information seeking, future research should examine the impact of online health discussions, network characteristics, and network interventions. This study can be used to further explore these relationships to inspire future hypothesis-driven studies and inform power calculations in the process.

Also, our sample size was small, and therefore the power to detect changes were limited. Only 7 noted an “other” gender category, 10 respondents were OPI, 41 stated they had a chronic disease, and 34 reported having a disability. Among the OPI population, none reported chronic disease. Although the information from this small sample can generally be used to help inform and direct public health campaigns, recognizing that these diverse populations have distinct cultures and histories that impact health risk and health information sharing patterns, it is critical for future research to include larger sample sizes to make disaggregation possible and to examine the unique needs of subpopulations that may be particularly vulnerable, such as young adults with disability or chronic illness who identify with a certain racial or ethnic group. 

## 5. Conclusions

The dissemination of health-related information through formal and informal social networks can refract the quality of information obtained. Modern digital interactions allow anyone to produce content as information disseminators, which was not as easily possible without higher technical skills in prior iterations of the web. In the midst of the pandemic, there have been cultural wars over COVID-19 guidelines (e.g., masking) and vaccines, and web 2.0 has allowed anyone a voice within the discussion. This has proved difficult for individuals to know who or what sources to trust; access to digital health information might not be enough. When examining factors connected to health and the decision-making process for health care, access to the internet does not always equate with higher digital HL [49], which relates to being able to evaluate the quality of digital sources of health information.

Given the vast reliance on online media for health information among young adults, there is a need to expand the frequency and sophistication of health interventions utilizing digital media and to enhance the trustworthiness of online content and digital health literacy [5,50]. Understanding young adults’ social networks and health information sources is important for designing effective health communications to reach all communities, especially those experiencing health inequities such as NH, OPI, and Filipino populations and for priority populations for equitable public health program planning. 

## Figures and Tables

**Table 1:** Descriptive breakdown in addition to percent with chronic disease status.

Variable		*n*	%	% With Chronic Disease
Full Sample		324	100	12.7
Gender	Male	98	30.2	8.2
	Female	219	67.6	15.1
	Other	7	2.2	0
Race/Ethnicity	NH	63	19.4	15.9
	OPI	10	3.1	0
	Japanese	48	14.8	16.7
	Chinese	20	6.2	20
	Other Asian	15	4.6	6.7
	Filipino	59	18.2	3.4
	African American ^1^	---	---	---
	Native American ^1^	---	---	---
	White	74	22.8	14.9
	Latino/a	18	5.6	16.7
	Refused	6	1.9	---
Disability	Yes	34	10.5	50
	No	290	89.5	8.3
Education Level	K-HS Dipl./GED	104	32.1	15.4
	Some College	94	29	6.4
	College Degree	122	37.6	15.6
	Don’t Know/Refused	4	1.2	---
Chance of Catching COVID-19	High	22	6.8	27.3
	Medium	101	31.2	19.8
	Low	178	54.9	8.4
	None	23	7.1	0
Perception of COVID-19 Affecting Life	Very sick	159	49.1	17.6
	A little sick	117	36.1	8.6
	Not a big deal to me	48	14.8	6.3
Health Literacy	Low	63	19.4	7.9
	High	261	80.6	13.8

^1^ Note: *n* < 10 not included for reported race/ethnicity categories.

**Table 2:** Cross-tabulation of socio-demographic and health literacy by age and social network descriptors.

Variable	Age (Years)		Number of People in Household		Number of People <18 Years in Household		Number of People >65 Years in Household		Network Size to Discuss Own Health		Network Size to Discuss Others’ Health	
	*n*	Mean (SD)	*n*	Mean (SD)	*n*	Mean (SD)	*n*	Mean (SD)	*n*	Mean (SD)	*n*	Mean (SD)
*Gender*												
Male	98	26.7 (5.73)	97	3.39 (1.64)	97	0.81 (0.99)	97	0.33 (0.75)	97	1.73 (1.82)	97	1.74 (1.80)
Female	219	26.3 (5.37)	215	3.89 (2.10)	215	0.86 (1.26)	215	0.27 (0.60)	218	2.11 (2.25)	216	2.31 (2.29)
Other	7	25.7 (5.94)	7	3.57 (1.27)	7	0.43 (0.54)	7	0.29 (0.76)	7	3.00 (3.51)	7	4.14 (5.40)
Total	317	26.4 (5.48)	319	3.73 (1.98)	319	0.84 (1.18)	319	0.29 (0.65)	322	2.00 (2.13)	320	2.13 (2.16)
*Race/Ethnicity*												
NH	63	25.7 (5.64)	63	3.97 (2.11)	63	1.25 (1.61)	63	0.16 (0.45)	63	1.83 (1.87)	63	2.32 (2.70)
OPI	10	21.2 (5.03)	10	6.40 (3.60)	10	0.60 (0.84)	10	0.70 (0.68)	10	1.00 (1.25)	10	1.80 (1.55)
Japanese	48	27.2 (5.81)	48	3.21 (1.49)	48	0.54 (0.80)	48	0.44 (0.82)	48	2.33 (2.26)	47	2.15 (1.91)
Chinese	20	26.5 (5.74)	20	3.50 (1.43)	20	0.25 (0.55)	20	0.35 (0.67)	20	1.80 (1.40)	20	2.05 (2.61)
Other Asian	15	28.4 (5.67)	15	3.27 (1.49)	15	0.13 (0.52)	15	0.20 (0.56)	13	2.69 (2.46)	14	2.43 (1.70)
Filipino	59	24.8 (4.99)	58	4.38 (1.61)	58	0.98 (1.02)	58	0.38 (0.77)	59	2.20 (2.53)	59	2.20 (2.03)
African American ^1^	---	---	---	---	---	---	---	---	---	---	---	---
Native American ^1^	---	---	---	---	---	---	---	---	---	---	---	---
White	74	28.0 (4.72)	71	3.08 (1.68)	71	0.83 (1.17)	71	0.18 (0.52)	74	1.95 (2.19)	72	2.00 (2.13)
Latino/a	18	25.8 (5.43)	18	3.67 (2.11)	18	0.94 (1.35)	18	0.11 (0.47)	18	2.50 (2.88)	18	2.56 (3.50)
Refused	6	28.3 (6.89)	6	3.67 (2.94)	6	0.67 (0.82)	6	0.33 (0.82)	6	1.33 (1.21)	6	1.83 (1.84)
Total	324	26.4 (5.48)	319	3.73 (1.96)	319	0.83 (1.17)	319	0.29 (0.65)	322	2.02 (2.16)	320	2.18 (2.28)
*Education Level*												
K-HS Dipl./GED	104	23.3 (5.36)	103	4.44 (2.23)	103	1.15 (1.29)	103	0.27 (0.58)	104	1.75 (1.98)	104	2.14 (2.39)
Some College	94	26.3 (5.01)	92	3.92 (1.88)	92	0.75 (0.95)	92	0.39 (0.76)	93	2.12 (2.45)	93	2.14 (2.14)
College Degree	122	29.1 (4.46)	120	2.93 (1.31)	120	0.57 (0.91)	120	0.21 (0.61)	121	2.23 (2.08)	119	2.29 (2.32)
Total	320	26.4 (5.49)	315	3.71 (1.92)	315	0.81 (1.08)	315	0.28 (0.65)	318	2.04 (2.17)	316	2.20 (2.28)
*Health Literacy*												
Low	63	25.8 (5.57)	62	3.98 (2.06)	62	0.94 (1.42)	62	0.26 (0.63)	62	2.42 (2.74)	61	2.30 (2.60)
High	261	26.6 (5.46)	257	3.67 (1.94)	257	0.81 (1.10)	257	0.29 (0.66)	260	1.92 (2.00)	259	2.15 (2.20)
Total	324	26.4 (5.48)	319	3.73 (1.96)	319	0.83 (1.17)	319	0.29 (0.65)	322	2.02 (2.16)	320	2.18 (2.28)

^1^ Note: *n* < 10 not included for reported race/ethnicity categories.

**Table 3:** Poisson regression model fit with age, gender, race/ethnicity (NH, Japanese, Chinese, Filipino, and White), chronic disease, pandemic perceptions (whether it would affect their life and chances of catching it), and health literacy as predictors of the number of people with whom respondents discussed their own health.

Predictors	Estimate	Standard Error	z Value	*p* Value	IRR	95% CI
Age	−0.002	0.008	−0.279	0.781	0.998	0.982, 1.014
Gender (Male ref)	0.135	0.100	1.349	0.177	1.144	0.943, 1.395
Native Hawaiian (Japanese ref)	−0.229	0.134	−1.714	0.087	0.795	0.611, 1.034
Chinese (Japanese ref)	−0.272	0.193	−1.411	0.158	0.762	0.515, 1.099
Filipino (Japanese ref)	−0.030	0.132	−0.230	0.818	0.970	0.748, 1.259
White (Japanese ref)	−0.102	0.131	−0.782	0.434	0.903	0.699, 1.168
Chronic Disease	0.131	0.124	1.052	0.293	1.140	0.888, 1.447
COVID-19 Affecting Life	0.225	0.173	1.301	0.193	1.252	0.904, 1.784
Chances of Catching COVID-19	0.371	0.091	4.094	<0.000	1.450	1.213, 1.732
Health Literacy	−0.204	0.107	−1.905	0.057	0.815	0.663, 1.010

Note: Male gender was used as the referent group for the gender variable; Japanese was used as the referent group for the race/ethnicity variable within the model.

**Table 4:** Poisson regression model fit with age, gender, race/ethnicity (NH, Japanese, Chinese, Filipino, and White), chronic disease, pandemic perceptions (whether it would affect their life and chances of catching it), and health literacy as predictors of the number of people respondents listened to in discussion of others’ health.

Predictors	Estimate	Standard Error	Z Value	*p* Value	IRR	95% CI
Age	0.005	0.008	0.608	0.543	1.005	0.989, 1.021
Gender (Male ref)	0.222	0.100	2.216	0.027	1.249	1.029, 1.525
Native Hawaiian (Japanese ref)	0.074	0.131	0.565	0.572	1.077	0.834, 1.394
Chinese (Japanese ref)	−0.080	0.186	−0.429	0.668	0.923	0.634, 1.319
Filipino (Japanese ref)	0.069	0.138	0.502	0.616	1.072	0.818, 1.406
White (Japanese ref)	−0.046	0.134	−0.340	0.734	0.955	0.735, 1.245
Chronic Disease	0.412	0.114	3.624	<0.000	1.510	1.203, 1.880
COVID-19′s Effect on Life	0.337	0.178	1.889	0.059	1.401	1.002, 2.021
Chances of Catching COVID-19	0.090	0.090	1.001	0.317	1.094	0.917, 1.305
Health Literacy	−0.037	0.112	−0.330	0.741	0.964	0.778, 1.206

Note: Male gender was used as the referent group for the gender variable; Japanese was used as the referent group for the race/ethnicity variable within the model.

**Table 5:** Descriptive breakdown by who did or did not search for COVID-19 or related information online and for whom in the past 4 weeks.

Variable		Total	No		Yes, for Me		Yes, for Others		Yes, for Me and Others	
		*n*	*n*	%	*n*	%	*n*	%	*n*	%
Full Sample		324	89	27	90	28	32	10	113	35
Gender	Male	98	30	30.6	27	27.6	8	8.2	33	33.7
	Female	219	57	26.0	62	28.3	23	10.5	77	35.2
	Other	7	2	28.6	1	14.3	1	14.3	3	42.9
Race/Ethnicity	NH	63	20	31.7	15	23.8	7	11.1	21	33.3
	OPI	10	4	40.0	1	10.0	3	30.0	2	20.0
	Japanese	48	8	16.7	15	31.3	3	6.3	22	45.8
	Chinese	20	2	10.0	8	40.0	1	5.0	9	45.0
	Other Asian	15	3	20.0	6	40.0	2	13.3	4	26.7
	Filipino	59	11	18.6	18	30.5	6	10.2	24	40.7
	African American ^1^	---	---	---	---	---	---	---	---	---
	Native American ^1^	---	---	---	---	---	---	---	---	---
	White	74	25	33.8	23	31.1	5	6.8	21	28.4
	Latino/a	18	8	44.4	2	11.1	4	22.2	4	22.2
	Refused	6	---	---	---	---	---	---	---	---
Disability	Yes	34	13	38.2	5	14.7	5	14.7	11	32.4
	No	290	76	26.2	85	29.3	27	9.3	102	35.2
Education Level	K-HS Dipl./GED	104	33	31.7	25	24.0	15	14.4	31	29.8
	Some College	94	26	27.7	23	24.5	12	12.7	33	35.1
	College Degree	122	28	23.0	42	34.4	5	4.1	47	38.5
	Don’t Know/Refused	4	---	---	---	---	---	---	---	---
Chronic Disease	Yes	41	10	24.4	11	26.8	5	12.2	15	36.6
	No	283	79	27.9	79	27.9	27	9.5	98	34.6
Health Literacy	Low	63	14	22.2	18	28.6	9	14.3	22	34.9
	High	261	75	28.7	72	27.6	23	8.8	91	34.9

^1^ Note: *n* < 10 not included for reported race/ethnicity categories.

## Data Availability

Data can be shared upon request.
